# Tropical Origin, Global Diversification, and Dispersal in the Pond Damselflies (Coenagrionoidea) Revealed by a New Molecular Phylogeny

**DOI:** 10.1093/sysbio/syae004

**Published:** 2024-01-23

**Authors:** Beatriz Willink, Jessica L Ware, Erik I Svensson

**Affiliations:** Department of Zoology, Stockholm University, Svante Arrhenius väg 18b, Stockholm 106-91, Sweden; Department of Biological Sciences, National University of Singapore, 14 Science Drive, Singapore 117558, Singapore; Division of Invertebrate Zoology, American Museum of Natural History, 200 Central Park West, New York, NY, 10024, USA; Department of Biology, Evolutionary Ecology Unit, Lund University, Sölvegatan 37, Lund 223-62, Sweden

**Keywords:** biogeography, extinction, graphical models, latitudinal diversity gradient, niche conservatism, paleogeography, speciation

## Abstract

The processes responsible for the formation of Earth’s most conspicuous diversity pattern, the latitudinal diversity gradient (LDG), remain unexplored for many clades in the Tree of Life. Here, we present a densely sampled and dated molecular phylogeny for the most speciose clade of damselflies worldwide (Odonata: Coenagrionoidea) and investigate the role of time, macroevolutionary processes, and biome-shift dynamics in shaping the LDG in this ancient insect superfamily. We used process-based biogeographic models to jointly infer ancestral ranges and speciation times and to characterize within-biome dispersal and biome-shift dynamics across the cosmopolitan distribution of Coenagrionoidea. We also investigated temporal and biome-dependent variation in diversification rates. Our results uncover a tropical origin of pond damselflies and featherlegs ~105 Ma, while highlighting the uncertainty of ancestral ranges within the tropics in deep time. Even though diversification rates have declined since the origin of this clade, global climate change and biome-shifts have slowly increased diversity in warm- and cold-temperate areas, where lineage turnover rates have been relatively higher. This study underscores the importance of biogeographic origin and time to diversify as important drivers of the LDG in pond damselflies and their relatives, while diversification dynamics have instead resulted in the formation of ephemeral species in temperate regions. Biome-shifts, although limited by tropical niche conservatism, have been the main factor reducing the steepness of the LDG in the last 30 Myr. With ongoing climate change and increasing northward range expansions of many damselfly taxa, the LDG may become less pronounced. Our results support recent calls to unify biogeographic and macroevolutionary approaches to improve our understanding of how latitudinal diversity gradients are formed and why they vary across time and among taxa.

One of the most striking features of species diversity is the non-uniformity of its global distribution. Tropical regions of the world generally have higher species richness compared to temperate regions, a pattern known as the latitudinal diversity gradient (LDG) (Hillebrand 2004). The LDG has fascinated naturalists from centuries, including both Darwin (1859) and Wallace (1876, 1878), who saw the climatic stability of the tropics as a factor hindering extinction and believed that stronger biotic interactions in the tropics could accelerate speciation. Wallace also suggested that climatic fluctuations, such as Pleistocene glaciations, were more pronounced in temperate regions, providing a limited opportunity for diversity to accumulate in these areas compared to the tropics (Wallace 1878). These early biogeographic hypotheses were further developed by ecologists and evolutionary biologists throughout the twentieth century (Dobzhansky 1950; Fischer 1960; MacArthur 1969), and today, the mechanisms responsible for the LDG remain a highly active area of research (e.g., Hanly et al. 2017; Rabosky et al. 2018; Gaboriau et al. 2019; Saupe et al. 2019; Meseguer and Condamine 2020).

Current hypotheses to explain the causes of the LDG can be broadly categorized into those that emphasize ecological effects on diversification rates versus those that emphasize time for speciation in different regions and the contingencies of evolutionary history (Pianka 1966; Mittelbach et al. 2007; Wiens 2011). At one end of this dichotomy, the tropics are portrayed as ecology-fueled species pumps, due to higher speciation rates (Wiens 2007), lower extinction rates (Weir and Schluter 2007; Pyron 2014), or both (Jablonski et al. 2006; Pyron and Wiens 2013; Rolland et al. 2014). The tropics have also been suggested to be the sources of most evolutionary novel lineages due to their greater geographical area (Rosenzweig et al. 1995; Belmaker and Jetz 2015), greater niche heterogeneity (Moritz et al. 2000; Salisbury et al. 2012), stronger biotic interactions (Dobzhansky 1950; Schemske et al. 2009), or higher mean temperature (Wright et al. 2006; Brown 2014). As Darwin and Wallace already anticipated, tropical diversity might also be buffered from the extinction that occurs in temperate regions because of greater climatic instability (Dynesius and Jansson 2000; Jablonski et al. 2006; Hawkins et al. 2007). In contrast, studies at the other end of the dichotomy have proposed that the amount of time available for diversification might be the predominant cause of variation in regional species richness, whether aligned or contrary to the LDG (Stephens and Wiens 2003; Stevens 2006; Economo et al. 2018; Miller and Román-Palacios 2021).

Understanding how ecological effects and historical contingencies shape regional diversification dynamics is, therefore, fundamental for a full understanding of the LDG, and it is crucial to critically evaluate the empirical evidence for these contrasting alternatives (Wiens 2011). However, regional biotas are not only shaped by varying speciation and extinction rates but also by dispersal (Salisbury et al. 2012; Smith et al. 2012; Antonelli et al. 2015; Jablonski et al. 2017). A lineage may disperse to a distant region with a similar biome, maintaining the LDG, or move to an ecologically novel area (i.e., a colder or warmer biome), thus altering the steepness of the LDG. Moreover, diversification and dispersal can act interdependently (Goldberg et al. 2011). For example, ecological limits to diversity, if present, could potentially reduce both speciation and incoming dispersal rates (Etienne et al. 2019). Similarly, ecological specialization and niche partitioning may result in fast speciation locally but trade-off with the evolution of dispersal ability (Jocque et al. 2010; Salisbury et al. 2012; Polato et al. 2018). Despite a potential key role of dispersal in molding the LDG, a prevailing focus on diversification dynamics may have curbed our understanding of the relative roles of each of these processes shaping regional biotas (Roy and Goldberg 2007).

To date, only a few studies have examined the combined effects of speciation, extinction, and dispersal in shaping diversity gradients (Antonelli et al. 2015; Rolland et al. 2015; Spano et al. 2016; Jablonski et al. 2017; Owens et al. 2017). The majority of these studies target clades with a relatively rich fossil record, which also enables process-based methods for inferring time-calibrated phylogenies, such as the fossilized birth–death process and total evidence approaches (Ronquist et al. 2012; Heath et al. 2014). However, most organisms, including insects, do not fossilize well (Wills 2001; Kidwell and Holland 2002), and therefore, other process-based methods are useful to calibrate molecular clocks and infer ancestral geographic ranges (e.g., Tiley et al. 2020). Biogeographic dating approaches have been long used as an alternative to fossil dating, but until recently, most of these methods relied on node calibrations (Kodandaramaiah 2011; De Baets et al. 2016) and have consequently failed to consider the joint probability of alternative diversification and dispersal scenarios (Ho et al. 2015).

Landis (2017) introduced a data-dependent generative model, in which speciation times and ancestral geographic states are jointly estimated over discretely defined epochs. Crucially, this approach used paleogeographical data to explicitly consider how the Earth’s dynamic past, through tectonic movements, has influenced the availability of dispersal routes for terrestrial animals. More recently, Landis et al. (2021) implemented a similar time-stratified approach to model within-biome dispersal and biome shifts, building on paleoecological literature and thus examining the role of phylogenetic niche conservatism in shaping biogeographic histories. These studies illustrate how empirical models of dispersal barriers can be used in lieu of fossils to implement process-based calibrations. They also show how time-dependent process-based models can be used to reconstruct ancestral diversity patterns, such as the LDG, while accounting for paleoclimatic changes. Here, we capitalized on these developments to investigate the phylogenetic and biogeographic history of a globally distributed group of insects, the damselfly superfamily Coenagrionoidea (Carle et al. 2008; Bybee et al. 2021).

Our target clade includes 2 families: the pond damselflies (family Coenagrionidae) and their close relatives, the featherlegs (family Platycnemididae). Coenagrionidae is a large family, distributed across all continents except Antarctica, and with species that are at extremes of trait and niche diversity. For instance, the family includes the tropical-rainforest phytotelmata-breeding helicopter damselflies, previously considered a separate family due to their morphological distinctiveness (Dijkstra et al. 2014; Toussaint et al. 2019). At the other extreme, Coenagrionidae also includes species restricted to Subarctic fens and marshes in the type genus *Coenagrion* (Paulson 2009, 2011). In the Nearctic alone, where most systematic niche data has been assembled, heterogeneity in habitat preference is striking. Species vary vastly in their affinities to forested areas, range sizes, and breeding habitats (Abbott et al. 2022). Platycnemididae, the other family in Coenagrionoidea, is composed by primarily lotic (i.e., inhabiting fast-moving water bodies) and paleotropical taxa, with some species reaching Northern Europe and East Asia (Boudot and Kalkman 2015; Dijkstra 2017).

Combined, the 2 families in Coeangrionoidea account for over a fourth of all Odonata species. Their ancient history (Kohli et al. 2021; Suvorov et al. 2022), rich ecological diversity (Abbott et al. 2022), and high dispersal potential as relatively large volant insects (May 2019) makes pond damselflies and featherlegs an excellent group to study the processes responsible for global diversity patterns, such as the LDG. Few studies have previously investigated the causes of the LDG in insects, suggesting important roles for speciation rate (Condamine et al. 2012), speciation time (Economo et al. 2018), and dispersal dynamics (Owens et al. 2017). Moreover, there is only one previous study that generated a dated species-level phylogeny for Coeangrionoidea, and it relied on phylogenetically unsupported taxonomy as backbone (Waller and Svensson 2017). There is, therefore, a need for an updated phylogeny of Coenagrionoidea with denser taxon sampling and for modern phylogenetic comparative analyses to uncover large-scale diversity patterns and their drivers in this ancient group of insects.

Here, we present the most extensively sampled phylogeny of pond damselflies and their relatives to date. We include over 35% of the approximately 1805 species, and thereby substantially increase the phylogenetic coverage of sampled taxa compared to previous studies (approximately 7% of all species in Dijkstra et al. 2014; and 23% in Waller and Svensson 2017). Recent phylogenomic studies have produced a robust and dated backbone for the order Odonata, which we used to inform the origin time, over 100 Ma, of the most recent common ancestor of pond damselflies and featherlegs (Kohli et al. 2021; Suvorov et al. 2022). We combine this root-age prior with an empirical paleogeographic model (Landis 2017), genetic data, and distribution range data of extant taxa, to simultaneously infer the divergence times and geographic distributions of ancestral lineages. We also use this time-calibrated phylogeny to quantify temporal and geographic patterns of diversification, and to jointly model how within-biome dispersal and biome shifts have shaped regional diversity throughout the history of the clade. Our results provide a comprehensive overview of the macroevolutionary history of this insect group and shed new light on the question of how speciation, extinction, and lineage movements influence global diversity patterns.

## Materials and Methods

Here, we summarize the main features of the phylogenetic analyses performed in this study. For a detailed description of data collection, sequence alignment, and the statistical approach used for biogeographic dating, diversification, and biome-shift analyses, see the Supplementary Material. We mention here the most relevant prior distributions. For complete information on priors and parameter proposals, see the Extended Methods and accompanying scripts. All novel sequence data used in this study is available through NCBI Genbank (Supplementary Table S2). The sequence alignment, distribution data, and code necessary to reproduce our results are available on Dryad (https://doi.org/10.5061/dryad.h18931znp). Phylogenetic analyses were partly conducted on the computer clusters at the LUNARC Center for Scientific and Technical Computing, Lund University, Sweden, and the Uppsala Multidisciplinary Center for Advanced Computational Science (UPPMAX).

### Sequence Data and Tree Topology Inference

We sampled a total of 669 taxa in the Coenagrionoidea superfamily, 556 of which are currently classified as Coenagrionidae and 113 as Platycnemididae (Supplementary Tables S1 and S2). In a recent phylogenomic study, the small family Isostictidae was recovered as sister to the clade including both Coeangrionidae and Platycnemididae (Bybee et al. 2021). As we did not sample Isostictidae, we hereafter use the superfamily name Coenagrionoidea to refer to Coenagrionidae (pond damselflies) and Platycnemididae (featherlegs) only. Samples were collected in the field (*n* = 142 species), or obtained from specimens in museum and private collections (*n* = 362 species), and publicly available data on NCBI (https://www.ncbi.nlm.nih.gov/genbank/) (*n* = 194 species). For each species, we obtained sequence data for up to 5 loci (cytochrome oxidase subunit I, 16S ribosomal DNA, histone 3, phenyl-methyltransferase, and 28S ribosomal DNA). Our molecular data consists of 1695 new sequences, 564 previously published sequences, and 105 unpublished sequences from the Naturalis Biodiversity Center, The Netherlands. We conducted all phylogenetic analyses using probabilistic graphical models and Bayesian inference for parameter estimation, using RevBayes v. 1.0.7, v. 1.0.12, and v. 1.1.1 (Höhna et al. 2014, 2016).

Our first analysis aimed to infer the topology of the Coenagrionoidea phylogeny. We used a rooted uniform tree prior (Ronquist et al. 2012) and a partitioned data scheme accounting for substitution rate variation among loci and among codon positions of coding sequences (Supplementary Table S3). We specified a General Time-Reversible model of molecular evolution for each partition and accounted for rate heterogeneity among sites by assuming gamma-distributed rates. Finally, we enforced the monophyly of each of the 2 families within Coenagrionoidea (Coenagrionidae and Platycnemididae), as supported by recent large-scale studies (Kohli et al. 2021; Bybee et al. 2021; Suvorov et al. 2022). To do this, we imposed a single topological constraint on the root node of the tree, which was necessary as preliminary analyses did not adequately resolve this well-established ancestral relationship. The maximum a posteriori probability (MAP) tree was used to summarize phylogenetic inference.

### Biogeographic Dating with Empirical Paleogeography

Next, we simultaneously inferred speciation times and ancestral distributions across the Coenagrionoidea tree using the empirical paleogeographic model and statistical approach developed by Landis (2017). Instead of relying on internal node calibrations, this approach jointly models ancestral dispersal and speciation events as conditional on historical shifts in oceanic barriers and the distribution ranges of extant taxa. To use biogeography in node-age estimation, we recorded species distributions from the literature, museum collections, IUCN Red List assessments, and reputable web sources for all extant taxa included in this study (Supplementary Table S4). Due to the low spatial resolution and coarse-grained nature of available data, species distributions were registered as presence or absence from administrative geographical regions (countries and states/provinces/territories for countries spanning multiple biogeographic areas; see below).

In the model developed by Landis (2017), dispersal can occur by 3 modes (short- medium- and long-distance), which are differentially limited by oceanic barriers. The empirical paleogeographic model captures historical changes in these water barriers due to continental drift (Landis 2017). Thus, paleogeography determines the probability of transitions between biogeographic states, for each dispersal mode and for each discrete time interval (epoch) since the maximum plausible root age of the tree. This time-heterogeneous process describing the probability of dispersal events in turn informs branch length estimation in absolute time (Landis 2017). For example, ancestral dispersal leading to a disjunct distribution in the present is inferred to occur with higher probability during time intervals (epochs) in which the currently disjunct land masses were adjacent, and hence connected by all 3 dispersal modes, than when they were separated by small or large water barriers, which would have then required medium- and long-distance dispersal, respectively.

**Figure 1.**
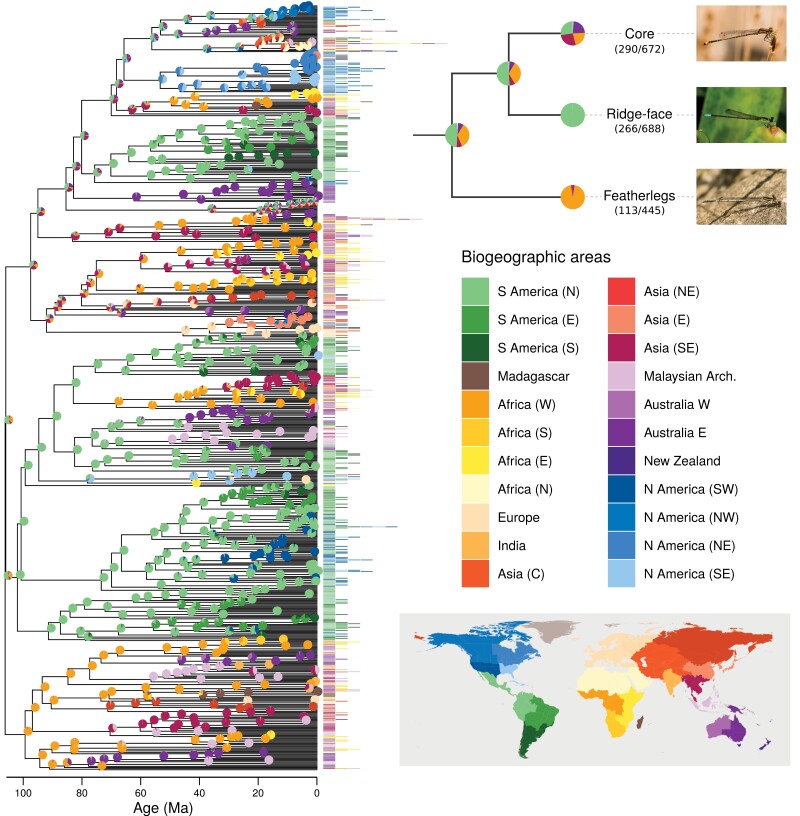
Inferring the biogeographic history of pond damselflies and their relatives the featherlegs (superfamily Coenagrionoidea). Speciation times and ancestral ranges were jointly estimated using a time-heterogeneous continuous-time Markov model, based on empirical paleogeography, an informed root-age prior and molecular and distribution data of 669 extant taxa. The empirical paleogeographic model in Landis (2017) was used to determine dispersal edges between 25 geographic areas across the globe (Greenland, Antarctica (E) and Antarctica (W) not shown in the color legend). Phylogenetic inference was summarized using a maximum a posteriori (MAP) tree. The distribution areas occupied by each of the extant taxa are shown at the tips of the tree. Pies show the proportion of each ancestral state in a random sample of 1000 posterior trees. The cladogram in the upper right shows the inferred ancestral ranges for the most recent common ancestor of Coenagrionoidea, and for each of the 3 major clades in the superfamily. The fraction of taxa sampled is shown in parenthesis. One representative species is illustrated for each clade: *Acanthagrion adustum* (‘core’), *Fredyagrion elongatum* (ridge-face’), *Platycnemis pennipes* (featherlegs). Photos: EIS.

Damselflies are aquatic insects with a terrestrial adult phase. They require fresh water habitats to breed and for larvae to develop (Corbet 1999). As damselflies cannot survive in or on salt water, it is highly likely that their dispersal has been historically constrained by oceanic barriers. Yet, Coenagrionoidea has a cosmopolitan distribution at present, ranging across all continents except for Antarctica and Greenland (Supplementary Table S4; Fig. 1). Unlike the larger dragonflies (suborder Anisoptera), in which long distance and sometimes trans-oceanic migration has been described (Wikelski et al. 2006; Anderson 2009; Troast et al. 2016), the majority of damselflies are small, weak fliers and tend to be more sedentary. For these biological reasons, we considered that a global biogeographic model of changes in dispersal routes due to continental drift would be a suitable approach to explore historical biogeography in these damselflies.

The empirical paleogeographic model in Landis (2017) consists of 25 biogeographic areas (Fig. 1; Supplementary Table S5) and 21 epochs between 240 Ma and the present (see Table S2 and Fig. S8 in Landis 2017). We combined this model with a root-age prior based on recent large-scale phylogenomic studies of major families in the whole order Odonata (Kohli et al. 2021; Suvorov et al. 2022). Suvorov et al. (2022) inferred a backbone phylogeny for Odonata using a supermatrix of 1603 gene orthologs and fossil calibrations with 20 crown fossil constraints. Kohli et al. (2021) used the sequences from nearly 3000 protein-coding genes and 17 fossil calibrations to reconstruct and date the phylogeny of Odonata. These studies date the origin of Coenagrionoidea to ~107 and ~116 Ma, respectively. We placed a normally distributed root-age prior with a mean of 120 and standard deviation of 20 to reflect this prior knowledge on the origin time of Coenagrionoidea. The normal distribution was truncated between 240 Ma, the approximate age of the most recent common ancestor (MRCA) of all Odonata (Misof et al. 2014; Suvorov et al. 2022), and 40 Ma, the minimum age according to the study with the youngest age estimate for Coenagrionoidea (Waller and Svensson 2017).

To assess the impact of our strongly informed root-age prior and the empirical paelogeographic model, we conducted 2 additional analyses to contrast our results. First, we ran an identical model but with a weakly informed root-age prior, in which the MRCA of Coenagrionoidea occurred with equal probability between 240 and 40 Ma. Second, we conducted an analysis with a uniform prior on the root age, in this case, bounded between 240 and 0 Ma, and ignoring the empirical paleogeographic data. This latter analysis hence excluded all empirical data (except the inferred origin time of Odonata) that could inform absolute divergence times and time-heterogeneous dispersal rates between biogeographic areas and was used to rule out the possibility that divergence times were influenced by artifacts arising from model construction. For a comparison of this paleogeographic dating approach to a dating analysis informed by fossil constraints and a pure-birth model, see the Supplementary Material.

### Diversification Through Time and Space

We quantified the temporal and geographic dynamics of diversification in Coenagrionoidea using the MAP tree from the dating analyses above. We used an episodic birth-death (EBD) model (Stadler 2011; Höhna 2015) to test for time-dependent diversification rate shifts since the MRCA of Coeangrionoidea. EBD analyses assume that diversification rates change through time in a piece-wise constant fashion, following a process of Brownian motion. Thus, for each epoch, rates are centered around the estimate in the previous epoch going backward in time (Höhna 2015; Magee et al. 2020). We used an EBD model with 20 equal-length time intervals and accounted for missing taxa in each of the main clades of Coenagrionoidea (Supplementary Fig. S4).

We used a Hidden State-Dependent Speciation and Extinction (HiSSE) model to estimate the diversification rates in 3 latitude-delimited biomes (tropical, warm-temperate and cold-temperate) while accommodating background diversification-rate heterogeneity across different branches of the tree (Beaulieu and O’Meara 2016). Heterogeneity in background diversification was taken into account by including a hidden trait with two states, which differed in their effects on diversification. We first compared the relative fit of this biome-dependent HiSSE model against a biome-independent model, which also accounted for diversification rate heterogeneity using a hidden trait with two states. We then conducted 3 biome-dependent diversification analyses under different data assumptions.

Of the 669 taxa in our phylogeny, 31 had an unknown biome state and were coded as ambiguous among the 3 biomes. Some of these taxa were identified at the generic level to wide-ranging genera (*n* = 12), and some had oceanic distribution ranges not covered by the paleobiome model used in the biome shift and within-biome dispersal analysis (*n* = 19, see below). An additional 99 species occupy ranges that extend across tropical and warm-temperate biomes (*n* = 46), across warm-temperate and cold-temperate biomes (*n* = 47), or across all 3 biome types (*n* = 6). We treated these taxa in 2 ways in separate analyses. In the main text, we present the results of our HiSSE model assigning taxa the coldest biome they inhabit. Because the common ancestor of Coenagrionoidea was tropical (see Results), biome states here indicate the extent to which a lineage has become established in a novel temperature niche. In the Supplementary Material, we show these results are qualitatively unchanged if we treat the widely distributed taxa as ambiguous between the biomes they currently occupy. Finally, we also conducted the biome-dependent HiSSE analysis using the Coenagrionoidea tree from the weakly informed dating analysis as input, and show these results are also qualitatively similar.

For all HiSSE analyses, biomes were assigned to each taxon based on present-day geography, using distribution data at the level of administrative areas (Supplementary Table S4), and biome reconstructions for the last 5 Ma (Jolly et al. 1998; Salzmann et al. 2008; Otto-Bliesner et al. 2020). We note that the HiSSE model is time-constant and does not consider how paleoclimatic change influences the geographic distribution of tropical and temperate biomes.

### Biome Shifts and Within-Biome Dispersal

To investigate the role of lineage movements on the formation and dynamics of the LDG, we jointly modeled dispersal events within biomes and biome shifts in Coenagrionoidea using the approach developed by Landis et al. (2021). Similar to our dating analysis, the likelihood of a dispersal event or biome shift is informed by the availability of suitable routes, but here, routes are potentially shaped by both land and biome continuity. For instance, if dispersal is limited by biome affinities, a lineage adapted to tropical rainforests in South East Asia should have a better chance of reaching Europe during periods of the Earth’s climatic history in which tropical rainforests were widespread in both regions, compared to more recent icehouse periods, when tropical rainforests had withdrawn from Europe. In contrast, if dispersal is unconstrained by ecological features, the same lineage movement would only depend on geographic continuity, which has been largely maintained between Europe and South East Asia throughout the last 100 Myr. Crucially, because the extent to which these geographic and ecological structures influence ancestral range shifts is unknown, Landis et al. (2021) treated them as free parameters that are estimated from the data as part of the biome shift and dispersal process (see Extended Methods).

As in our dating analysis, the dynamics of biome availability and connectivity are represented in this model by time-dependent graphs, informed by literature data. Here, we build on the paleobiome graphs used in Landis et al. (2021), which include 3 broad biome categories (tropical, warm-temperate, and cold-temperate) across 6 geographic regions (Southeast Asia, East Asia, Europe, South America, Central America, and North America), and eight time intervals over the last 106 Myr (Supplementary Table S7). We focus on the last 106 Myr, based on the inferred origin time of Coenagrionoidea in our dating analysis (see Results). We nonetheless expand the empirical model of Landis et al. (2021) to accommodate more of the global distribution of Coenagrionoidea, by adding 3 geographic regions (Africa, Australia, and India) that harbor a large fraction of pond damselfly and featherleg diversity (Supplementary Figs. S2–S3). Species with distributions outside these main geographic regions (e.g., Pacific islands and Central Asia endemics, *n* = 32) were treated as missing data (i.e., included in the tree but lacking a biome-region state). Similarly, extant species with ranges that span 2 or more geographic regions or biomes (*n* = 151) were treated as ambiguous between their current states (e.g., a tropical rainforest species ranging from Central America to South America was coded as ambiguous between the Tropical+SAm and Tropica+CAm states, see also Supplementary Table S7).

We used stochastic character mapping to sample lineage proportions through time and evolutionary histories in the dispersal and biome shift model. Following Landis et al. (2021), we also used these sampled histories to quantify the frequency of events (biome shifts and region shifts) and event series (see below) throughout the evolution of Coenagrionoidea. *Event series* represent different patterns of consecutive transitions between biomes and regions (Supplementary Table S8). First, we quantified instances in which both biomes and regions shifted in a lineage and recorded whether the biome shift preceded the dispersal event (*biome-first* series) or *vice versa* (*region-first* series). Then, we examined consecutive changes in biomes and regions separately. A *reversal* corresponds to a series of events with the same starting and ending state. For instance, a *biome reversal* occurs when a tropical species shifts to a warm-temperate biome and then back to the tropics. A *flight* is a series of 2 events across 3 different states. For example, a lineage that originates in Africa and then disperses to India and South East Asia would represent a *region flight*. We compute and report the proportion of each event and *event series* type across our posterior sample of evolutionary histories (see Extended Methods).

In this and previous analyses, we report the posterior mean (PM) and 95% highest posterior density (HPD) intervals for parameter estimates. For proportions of events and *event series*, we also report 80% HPD intervals. Phylogenetic inferences and parameter estimates were summarized using R (R Core Team 2021). Plots we produced with the R packages *ggplot2* (Wickham 2016) and *ggtree* (Yu et al. 2017).

## Results

### Topology Inference

The relationships reconstructed here (Supplementary Figs. S5-S8) are largely congruent with previous works (Dijkstra et al. 2014; Toussaint et al. 2019; Bybee et al. 2021). We fixed the relationship between the two families of Coenagrionoidea, Platycnemididae (featherlegs) and Coenagrionidae (pond damselflies) as resolved in previous genomic-scale backbone phylogenies (Suvorov et al. 2022; Bybee et al. 2021; Kohli et al. 2021). Within Platycnemididae, our tree recovers the previously well-supported relationships between the subfamilies Platycnemidinae, Disparoneurinae and Calicnemiinae, while the phylogenetic placement of Allocnemidinae, Idiocnemidinae and Onychargiinae (sensu Dijkstra et al. 2014) remains elusive across studies (Supplementary Fig. S5a). Nonetheless, the generic composition of each of these subfamilies (Supplementary Fig. S6) is consistent with Dijkstra et al. (2014), who sampled a relatively large fraction (~10%) of all featherleg species.

Within Coenagrionidae, the relationships between 4 main clades, the ‘core’ pond damselflies (sensu Dijkstra et al. 2014), the genus *Argia* (Rambur, 1842), the subfamily Protoneurinae (sensu Dijkstra et al. 2014) and the strict-sense “ridge-face” clade (Dijkstra et al. 2014), have remained contentious among recent studies (Supplementary Fig. S5b). Our results are congruent with the most recent backbone phylogeny for Odonata, which used targeted genomics to resolve ancestral relationships in the order (Bybee et al. 2021) (Supplementary Fig. S5b). In both phylogenies, *Argia* and the strict-sense “ridge-face” pond damselflies form a clade, in turn sister to Protoneurinae (Supplementary Fig. S5b). We hereafter refer to this more inclusive clade as “ridge-face” pond damselflies to distinguish it from the “core” clade identified in Dijkstra et al. (2014).

Three large and well-sampled genera of “core” pond damselflies (*Ischnura*, *Enallagma*, and *Acanthagrion*) form a clade that is sister to *Agriocnemis* (incl. *Mortonagrion* and *Argiocnemis*, see Extended Results). We recovered these previously supported relationships in our phylogenetic inference (Supplementary Fig. S5c). We also recovered a close relationship between *Coenagrion* and *Pseudagrion* (and allied genera) relative to the clade described above and consistent with previous studies (Supplementary Fig. S5c). However, our results differ from previous phylogenies in that they place other relatively small South Pacific genera (*Xanthocnemis*, *Austrocoenagrion*, and *Stenagrion*) within the same clade as *Coenagrion* and *Pseudagrion*, albeit with low support (Supplementary Fig. S5c).

Finally, in the strict-sense “ridge-face” pond damselflies (i.e., excluding *Argia* and Protoneurinae), relationships between the most distinct genera are also largely congruent among studies. The neotropical *Telebasis* and the paleotropical *Ceriagrion* form a clade separately from the strict rainforest dwellers *Mecistogaster*, *Metaleptobasis*, and *Teinobasis* (Supplementary Fig. S5d). In both our study and in Toussaint et al. (2019), who focused on ridge-face pond damselflies, the neotropical genus *Metaleptobasis* and the Neotropical helicopter damselflies (including *Mecistogaster*) show closer affinity, while in Dijkstra et al. (2014), *Mecistogaster* and the paleotropical *Teinobasis* appear more closely related (Supplementary Fig. S5d).

Out of 115 genera included in this study, 45 are represented by single species, either because the genus is monotypic (*n* = 22, i.e., having only one described species) or because the material was available for only one species (*n* = 23). Of the remaining 70 genera, 50 were recovered as monophyletic, in most cases with high posterior probability (Supplementary Table S9; Supplementary Fig. S9; median = 1.00, min = 0.55, max = 1.00). A total of 20 genera were thus recovered as paraphyletic (Supplementary Table S9). Of these, 5 pose phylogenetic relationships that challenge current taxonomy supported by morphology (*Acanthagrion*, *Cyanallagma*, and *Oxyagrion*) or earlier molecular studies (*Erythromma*, *Platycnemis*), 10 are supported by recent studies that also inferred paraphyly (*Agriocnemis*, *Coeliccia*, *Elattoneura*, *Indocnemis*, *Ischnura*, *Mecistogaster*, *Nesobasis*, *Pseudagrion*, *Prodasineura*, and *Teinobasis*), and 5 lack previous studies with taxonomic sampling that would enable detection of paraphyly (*Aciagrion*, *Enallagma*, *Mortonagrion*, *Psaironeura*, *Telebasis*). We discuss these potentially paraphyletic genera in the Extended Results (Supplementary Figs. S10-S25).

### Dated Phylogeny and Ancestral Biogeography

Our biogeographic dating analysis using a strongly informed root calibration returned a mean age estimate for the MRCA of Coenagrionidae and Platycnemididae of 105 Ma (95% HPD interval = 64–145 Ma; Supplementary Fig. S26). In contrast, applying a broad uniform prior to the root age resulted in a much younger estimate of 67 Ma (95% HPD interval = 40–118 Ma; Supplementary Fig. S26). An analysis excluding empirical paleogeographic data produced, as expected, a flat posterior root age distribution between 240 and 0 Ma (Supplementary Fig. S26). Finally, an analysis excluding paleogeographic data and relying on fossil constraints for node-dating returned a similar root-age estimate as the strongly informed biogeographic dating analysis (Supplementary Table S10). Relatively shallow nodes (i.e., at the level of genera) had older age estimates in the strongly informed biogeographic dating analysis than in previous studies of comparable clades (Supplementary Table S10). In contrast, our fossil-based approach generally resulted in young age estimates for shallow nodes compared to other large-scale studies and in agreement with genus-level studies (Supplementary Table S10). Despite large dating differences between the two biogeographic analyses in this study, biogeographic trends were qualitatively similar between strongly informed and weakly informed models (Supplementary Figs. S27–S33). Here, we focus on results under the strongly informed prior, and present the full results under the weakly informed prior in the Supplementary Material.

Extant pond damselflies and featherlegs are globally distributed (Fig. 1). Our ancestral biogeographic inference indicated that Coenagrionoidea most likely originated in either Northern South America or Western Africa (Fig. 1; Supplementary Fig. S27), as these two ancestral ranges combined accounted for 88% of the posterior samples of the root state. The two extant lineages descending from this common ancestor had contrasting biogeographic histories. The featherlegs (family Platycnemididae) originated in Western Africa (PS = 0.959; Fig. 1; Supplementary Fig. S27) and likely dispersed throughout the Old World after multiple departures from Africa (Fig. 1; Supplementary Figs. S28–S29). The pond damselflies (family Coenagrionidae) are in turn composed of 2 clades with distinct dispersal dynamics. The “ridge-face” clade of pond damselflies originated with high probability in Northern South America (PS = 0.999; Fig. 1; Supplementary Fig. S27) and thereafter remained largely neotropical, with a few successful dispersal events to the Nearctic (in *Argia* and *Nehalennia*), tropical Africa and Asia (in *Ceriagrion*) and presumably also across the Pacific (*Melanesobasis*, *Teinobasis*, *Amphicnemis* and allied genera) (Fig. 1; Supplementary Figs. S30–S31). Finally, the “core” pond damselflies have a much more dynamic dispersal history, particularly in the genus *Ischnura* (Fig. 1; Supplementary Figs. S32–S33), and currently occupy all available biogeographic areas and many oceanic islands not considered in this analysis (e.g., *Megalagrion* in Hawaii; Supplementary Figs. S32–S33). While the origin of “core” pond damselflies is also tropical, the geographic range of their MRCA remained elusive (Fig. 1; Supplementary Fig. S27).

### Temporal Dynamics of Diversification

**Figure 2.**
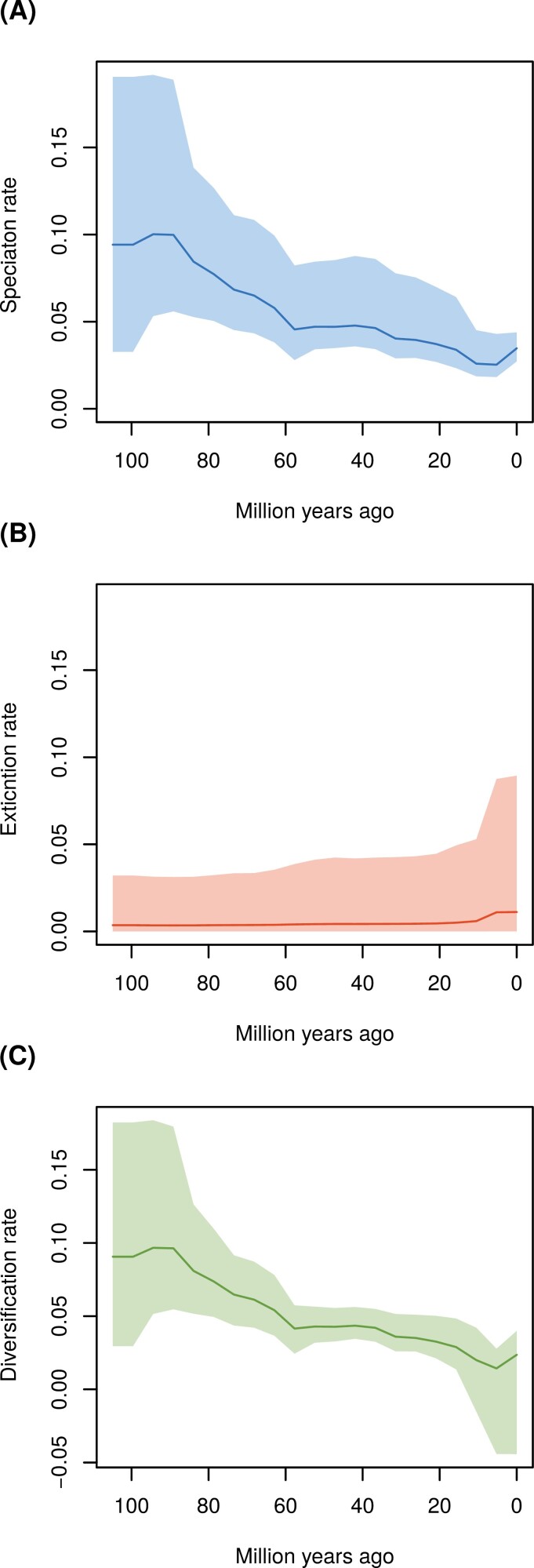
Diversification rate changes since the MRCA of Coenagrionoidea: (a) speciation, (b) extinction, and (c) net diversification. Diversification rates were estimated on the maximum a posteriori (MAP) tree from the biogeographic phylogenetic dating analysis using a root age calibration based of previous phylogenomic studies (Kohli et al. 2021; Suvorov et al. 2022). Changes in diversification rates were modeled under an episodic birth-death (EBD) process, over 20 equal-length time intervals and assuming autocorrelation among consecutive time intervals (see Extended Methods).

Our EBD analysis showed a decline in the rate of speciation since the origin of Coenagrionoidea, particularly between 85 and 55 Ma (Fig. 2a). This analysis also detected a recent but modest increase in extinction (Fig. 2b). The combined effects of speciation and extinction dynamics resulted in a relatively constant net diversification rate in the early history of Coenagrionoidea, followed by a period of declining diversification (~85–55 Ma) and another period of constant to slowly declining diversification (~55 Ma to the present) (Fig. 2c). As a result of increasing extinction over the last 5 Myr, the 95% HPD interval of diversification over this period includes the possibility of net diversity loss near the present (Fig. 2c). EBD dynamics modeled on the dated phylogeny under a weakly informed root-age prior are qualitatively similar to the patterns described above (Supplementary Fig. S34).

### Biome-Dependent Diversification

**Figure 3.**
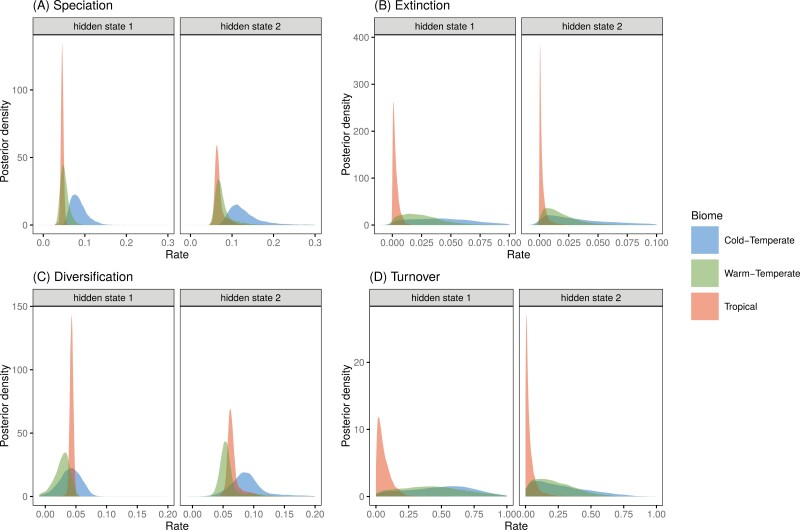
Latitudinal effects on diversification of pond damselflies and their relatives the featherlegs (superfamily Coenagrionoidea). (a) Speciation and (b) extinction rates at tropical and temperate latitudes were estimated using a Hidden-State Dependent Speciation and Extinction (HiSSE) model that accounts for background heterogeneity in diversification rates across the phylogeny, by including a hidden trait with two states. (c) Diversification was calculated as the net difference between speciation and extiction and (d) turnover was calculated as the ratio between extinction and speciation. The histograms show the posterior distribution of parameter estimates.

The biome-dependent HiSSE model was very strongly supported over an alternative model with only biome-independent rate heterogeneity (Supplementary Table S11). As in the paleogeographic dating analysis, the HiSSE model inferred a tropical distribution with high certainty for the MRCA of pond damselflies and featherlegs (Supplementary Fig. S35). Following dispersal from the tropics, both speciation and extinction were accelerated in cold-temperate biomes, while warm-temperate biomes prompted relatively low speciation and high extinction (Supplementary Table S12; Fig. 3a,b). As a result, net diversification rates were similar across biomes (Supplementary Table S12; Fig. 3a,b). In contrast, lineage turnover occurred at a faster pace in both temperate biomes compared to the tropics (Supplementary Table S12; Fig. 3d).

Background diversification rates distinguished the “core” pond damselflies and the “ridge-face” genera *Argia* and *Ceriagrion*, all with relatively high diversification, from the remaining “ridge-face” pond damselflies and the featherlegs, with relatively low diversification (Supplementary Fig. S35). Results of the HiSSE analysis using the MAP tree under a weakly informed root-age prior and using a character matrix that included ambiguous states for wide-ranging taxa were qualitatively similar (Supplementary Tables S13–S14; Supplementary Figs. S36–S39). A model run without data recovered prior distributions, as expected (Supplementary Fig. S40).

### Dispersal and Biome Shift Dynamics

**Figure 4.**
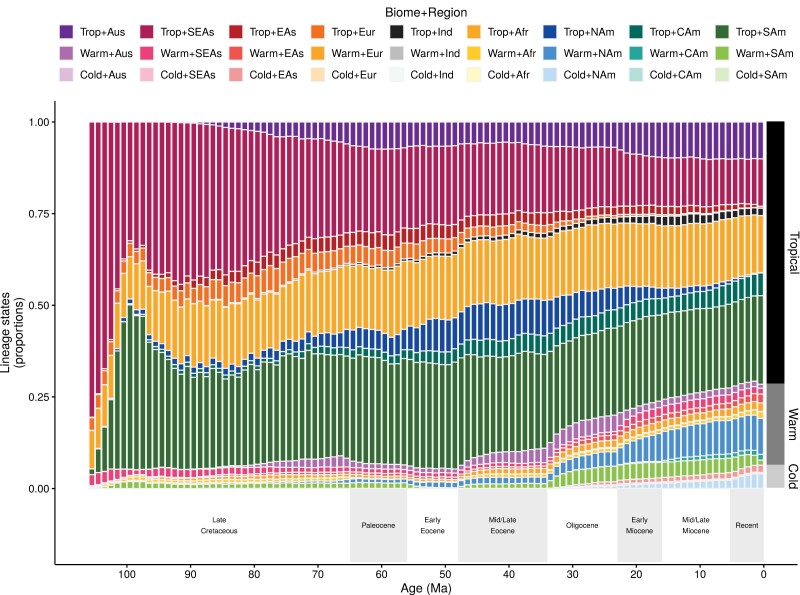
Proportion of Coenagrionoidea lineages in each biome-region state and through time. Lineage proportions were computed from stochastic histories sampled in a time-heterogeneous model of biome-region shifts (Landis et al. 2021). The dark-gray to light-gray bars on the right show the proportions of the 3 biome states in extant taxa sampled in this study (669 out of ~1800 species).

Estimated weighing parameters for geographical (w_G_ = 0.003) and biome features (w_B_ = 0.975), indicated that lineage movements in Coenagrionoidea are strongly influenced by biome affinities, while land connectivity independently of ecological features has a marginal effect on shaping ancestral ranges. It is thus not surprising that ancestral state reconstructions differed somewhat from those in our biogeographic dating model, which considered paleogeographic but not paleoecological history. In both models, we obtained strong support for a tropical ancestor of pond damselflies and featherlegs (Figs. 1 and 4; Supplementary Fig. S41). However, unlike the biogeographic dating model, the ancestral region with highest posterior probability in the biome-shift model was South East Asia (Fig. 4; Supplementary Fig. S41). Featherlegs and “core” pond damselflies were also inferred as most likely originating in tropical South East Asia, in contrast to the biogeographic dating analysis, while “ridge-face” pond damselflies originated with the highest posterior probability in tropical South America, consistent with the biogeographic dating analysis (Supplementary Fig. S41).

Clade-wide biome-region dynamics over time point to 3 major themes in the ancestral distribution of Coenagrionoidea. First, lineage diversity has been overwhelmingly tropical throughout history, with a rapid increase of taxa in South America shortly after the origin of the clade (Fig. 4). Second, a cooling trend during the Oligocene coincided with a slow and steady increase in temperate diversity, which continues to the present and is most notable in warm-temperate North America (Fig. 4). Finally, inference of relative biome-shift rates suggested a dynamic diversity exchange between tropical and warm-temperate biomes, indicated by a moderate rate of dispersal from tropical to warm-temperate biomes and fast dispersal back to the tropics (Supplementary Fig. S42). In contrast, cold-temperate biomes gain diversity and lose diversity at comparable and slow rates (Supplementary Fig. S42).

**Figure 5.**
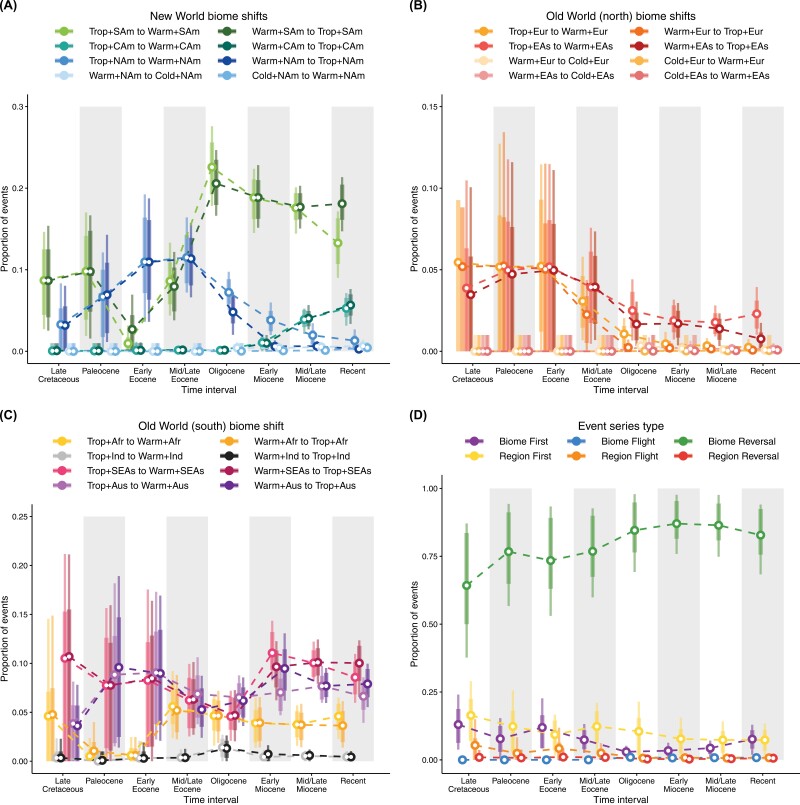
Proportion of biome-shift events (a–c) and event series (d) through time. Event series are defined as two consecutive state shifts, for instance, two dispersal events, two biome shifts, or one type of event followed by the other. Niche conservatism predicts an abundance of *biome reversals*, where lineages return to ancestral climatic niches, compared to *biome flights*, in which lineages shift towards increasingly colder or warmer biomes. The proportion of biome versus region series suggest that biome shifts within regions dominate the biogeographic history of Coeanogrionoidea, whereas long-distance dispersal within biomes is relatively rare. Finally, *Region-first* and *Biome-first* series summarize biogeographic histories that involve both dispersal and biome shifts. The proportions of these series indicate whether dispersal to a new region precedes adaptation to a new biome (*Region-first*) or *vice versa* (*Biome-first*). While both types of events are relatively rare, the cooling trend that started in the mid/late Eocence marked a decline in the proportion of *Biome-first* events. Event proportions were estimated from stochastic histories sampled in a time-heterogeneous model of biome-region shifts. Circles represent posterior means, bars represent 80% HPD intervals, and lines represent 95% HPD intervals.

The dynamic diversity exchange between tropical and warm-temperate biomes also emerged within regions, but the timing of these events seems to have varied across the planet. In South America, increased shifts between tropical and warm-temperate biomes followed the Oligocene cooling trend (Fig. 5a), as warm-temperate biomes became more available in the region (Supplementary Figs. S2–S3). In North America and Northern Eurasia (Fig. 5a,b), the exchange occurred mainly before the end of the Eocene, after which tropical biomes began to decline in these regions (Supplementary Figs. S2–S3). Finally, in the rest of Afroeurasia (Africa, India, and South East Asia) and in Australia, shifts between tropical and warm-temperate biomes were comparatively stable over time (Fig. 5c).

We note that stochastically mapped character histories were largely congruent with regionally available biomes (Supplementary Fig. S43, see also Extended Methods). The largest proportion of lineages with mismatched states (i.e., lineages with biome states that are regionally unavailable) occurred at the start of the Miocene, when nearly 6% of Coenagrionoidea lineages were inferred to occupy tropical biomes in North America (Fig. 4), but tropical biomes in this region became marginal during the transition between the Oligocene and Miocene, in the empirical paleobiome model (Supplementary Figs. S2–S3). As the proportion of tropical lineages in North America decreased during the Miocene (Fig. 4), congruence between inferred and available states increased (Supplementary Fig. S43).

Consistent with estimates of biome-shift rates (Supplementary Fig. S42) and relatively frequent shifts between tropical and warm-temperate biomes within regions (Fig. 5a–c), we found that *biome reversals* (e.g., from tropical to warm-temperate and back to tropical) are by large the most frequent *event series* across Coenagrionoidea (Fig. 5d). In contrast, *region reversals* and *biome flights* are extremely rare, while *region flights* and series involving both biome shifts and dispersal events occur at intermediate frequencies (Fig. 5d). Interestingly, the proportion of *region flights* dropped to almost zero during the Oligocene (Fig. 5d), when our empirical paleobiome model encodes a sharp decline in the continuity of tropical biomes across the Earth (Supplementary Figs. S2–S3). Together, *event series* reconstructions further indicate that movements within relatively warm areas of the globe (i.e., tropical and warm-temperate biomes) are the most prominent features in the historical biogeography of Coenagrionoidea.

## Discussion

In this study, we used a combination of biogeographic and multi-locus sequence data from extant taxa, empirical paleogeographic and paleobiome models (Landis 2017; Landis et al. 2021), and dating priors from recent phylogenomic analyses (Kohli et al. 2021; Suvorov et al. 2022). Our goal was to understand how biome-dependent diversification and dispersal shaped the current latitudinal diversity gradient in the most speciose superfamily of damselflies. While inferences about the age of Coenagrionoidea are notably sensitive to prior information (Supplementary Fig. S26), our ancestral biogeographic reconstructions based on empirical paleogeography are robust to node-age uncertainty (Supplementary Figs. S27–S33). Biogeographic inferences that additionally consider ecological niche conservatism, by modeling the effects of biome continuity on lineage movements, were partly in conflict with the paleogeographical-only model, especially in deeper nodes of the tree. Nonetheless, all of our analyses concurred on a tropical origin of Coenagrionoidea, followed by rapid dispersal throughout the world’s tropics, and more recently into high latitudes.

Despite their early global presence, diversification rates in Coenagrionoidea have declined since their evolutionary origin (Fig. 2). Diversification dynamics also vary between biomes, with cold-temperate lineages undergoing the fastest speciation and extinction, but with limited consequences for net diversification (Fig. 3). In contrast, biome-shifts, via lineage movements and paleoclimatic change, have resulted in a growing representation of temperate lineages, particularly as the Earth’s climate became cooler during the Oligocene (Fig. 4). Taken together, our results point to an early start of tropical diversification as the main driver of the LDG in Coenagrionoidea. Diversity gains due to biome shifts and accelerated speciation in temperate regions have counteracted the LDG, yet these effects may be at least partially swamped by a tendency of damselfly lineages to stay in relatively warm areas of the planet and a relatively fast pace of extinctions outside the tropics.

Our topology inference of Coenagrionoidea recovered phylogenetic relationships that are largely congruent with other recent molecular phylogenetic studies (Dijkstra et al. 2014; Toussaint et al. 2019; Bybee et al. 2021). However, our study also calls for the revision of multiple generic classifications based exclusively on morphological evidence and for further phylogenetic studies of a few particularly understudied clades (see Extended Results). We note that the root node of the tree, which marks the divergence between the pond damselfly and featherleg families was not resolved in our preliminary analysis, despite being strongly supported in multiple phylogenomic studies (Kohli et al. 2021; Suvorov et al. 2022; Bybee et al. 2021). In our preliminary analysis, the featherlegs were recovered as a monophyletic group, but their relationships to the main clades within the pond damselflies were uncertain. We, therefore, imposed a topological constraint to enforce the monophyly of each family in our final phylogenetic analysis. This resulted in strongly supported relationships between pond damselfly clades, in agreement with the most comprehensive backbone phylogeny of Odonata to date (Supplementary Fig. S5c, Bybee et al. 2021). Nonetheless, enforcing the monophyly of pond damselflies may have also contributed to underestimating branch lengths at the base of the family (see Supplementary Table S10). Ancestral introgression and incomplete lineage sorting in the early history of Coenagrionoidea are likely causes for phylogenetic discordance, and their effects tend to be aggravated in analyses based on concatenated loci (Kubatko and Degnan 2007). Moreover, both processes have been pervasive in the deep history of Odonata, leaving a genetic footprint within and between damselfly superfamilies (Suvorov et al. 2022).

Our biogeographic dating analysis, combining empirical paleogeography with prior information on the root age of Coenagrionoidea, resulted in an estimated time for the MRCA of 105 Ma (Supplementary Fig. S26). This estimate is only slightly younger, yet more uncertain, than the age of Coenagrionoidea in the phylogenomic studies used for calibration (Kohli et al. 2021; Suvorov et al. 2022). Our estimates of internal node ages under the strongly informed prior are also consistent with most fossil records, except for specimens attributed to Platycnemididae, which have been dated to ~99 Ma (Zheng et al. 2017), before the inferred origin of crown Platycnemididae in this analysis (~80 Ma) and in previous studies (Supplementary Table S10). Compared to shallow (genus-level) nodes dated in previous studies, inferred ages in this analysis are relatively older (Supplementary Table S10). In contrast, the dating analysis with a weakly informed root-age prior, returned a younger origin time for Coenagrionoidea (Supplementary Fig. S26), which was also younger than estimates in previous studies (Supplementary Table S10). However, this latter analysis produced age estimates for shallow nodes that were usually closer to estimates in previous studies (see, for example, *Ischnura*, *Enallagma*, *Nesobasis*, *Nehalennia*, and *Platycnemis* in Supplementary Table S10).

These results underscore the challenges in dating phylogenies of groups of organisms with an incomplete fossil record and the limitations of simple models of dispersal and paleogeography. The statistical approach developed by Landis (2017) is appealing for its ability to jointly sample dispersal histories and diversification events. However, the computational challenges to the likelihood calculation over a large space of states currently prohibits incorporating critical complexity into the paleogeographic model (Ree and Sanmartín 2009). A terrestrial graph at a global scale seems suitable for widely distributed damselflies, which require fresh water habitats to breed (Corbet 1999). Nonetheless, many genera of pond damselflies are endemic to specific archipelagos, while some taxa are widely distributed across continents (Fig. 1; Supplementary Table S4). In the present analysis, island endemics are subsumed into larger biogeographic areas or coded as missing data (see Supplementary Material), and distribution ranges spanning 2 or more areas are treated as character-state uncertainty in extant taxa. A model that incorporates branch heterogeneity in dispersal rates and captures the more fine-grained paleogeographic events that shaped the distribution of island endemics would likely improve biogeographic dating in this and other diverse groups of insects that vary in their ease of dispersal (e.g., Jones et al. 2016; Landis et al. 2018) and tend to leave a relatively sparse fossil record (Wills 2001). Yet, such extensions, as well as the use of large genomic data sets, under a fully Bayesian approach, are currently constrained by computational limitations.

Despite these challenges to node dating, both biogeographic dating analyses under alternative root-age priors indicate that the MRCA of pond damselflies and featherlegs originated in either Northern South America or Western Africa (Fig. 1; Supplementary Fig. S27). These areas were separated by narrower water barriers at the presumed origin of Coenagrionoidea (particularly under the strongly informed root-age prior) and have preserved tropical rainforests throughout the Cenozoic, notwithstanding climatic change (Morley 2007; Couvreur et al. 2011). In contrast, the biome-shift and dispersal analysis inferred the ancestral range of Coenagrionoidea in tropical South East Asia with relatively high (>80%) posterior probability (Supplementary Fig. S41). Two other deep nodes in the Coenagrionoidea phylogeny, the MRCAs of the featherlegs and “core” pond damselflies, were also incongruent between models and reconstructed with a more likely distribution in South East Asia according to the biome-shift and dispersal model (Fig. 1; Supplementary Fig. S41). Shallower nodes and nodes within the “ridge-face” clade of pond damselflies were generally more consistent between models (Fig. 1; Supplementary Fig. S41).

Conflicting inferences of ancestral geographic ranges are most notable for clades with a geographically widespread distribution across the Australia and the Old World and a historical affinity to tropical biomes. For example, some clades, such as the featherleg subfamily Disparoneurinae (including *Elattoneura*, *Prodasineura*, and *Nososticta*) and the large ‘core’ Coenagrionidae genus *Pseudagrion* are currently distributed between tropical Africa, India, SE Asia and Australia (Supplementary Figs. S28 and S32). In the paleogeography-only analysis, the assumption of rare long-distance dispersal likely drove the origin of Coenagrionoidea to either of 2 species-rich areas, Western Africa and Northern South America, that were connected by medium-distance dispersal in the early history of the clade. Thereafter, lineages with an austro-paleotropical distribution were more likely to descend from a Western African ancestor. Meanwhile, the empirical features of the biome-shift and dispersal model assumed an extent of tropical biome contiguity across austro-paleotropical regions, particularly during the warm Eocene, when SE Asia (including the Malaysian Archipelago) bridged tropical biomes in Africa and India to tropical biomes in Australia (Supplementary Figs. S2 and S3). Consequently, this model favored a scenario where austro-paleotropical lineages originated in tropical SE Asia and then dispersed to other regions as biome corridors formed over the course of continental drift.

Our results thus highlight the sensitivity of ancestral range reconstructions to assumptions embedded in dispersal graphs, and, specifically the relative importance of physical barriers versus niche conservatism in restricting dispersal events. The biome-shift and dispersal model allowed for either geographical or ecological features to dominate dispersal histories, by implementing weighing parameters for the different types of adjacency matrices (Landis et al. 2021). Our results strongly emphasized the role of biome contiguity and availability over merely land contiguity in shaping the biogeographic history of Coenagrionoidea. Therefore, the discrepancy in ancestral range inferences may have arisen from oversimplifying dispersal in the paleogeography-only model, by ignoring whether distant regions share ecological features. Biogeographic patterns across North America and Europe, such as post-glaciation range expansions, further support the importance of (tropical) niche conservatism in damselflies (Heiser and Schmitt 2010; Corser et al. 2014), arguing for the inclusion of biome affinities in dispersal models at a global scale. Nonetheless, identifying which tropical region (South America, Western Africa, or SE Asia) is the cradle of Coenagrionoidea will likely require a better understanding of long-distance movements to inform dispersal models in a phylogenetic framework (Jønsson et al. 2016).

The early evolution of Coenagrionoidea was characterized by fast diversification, followed by a decline in the rate of accumulation of new species (Fig. 2). This is a common feature of dated phylogenies (Morlon et al. 2010), which has been subject to considerable controversy and which can be attributed to several possible causes including statistical artifacts (reviewed in Moen and Morlon 2014). One possibility is that regional diversification slows down over time because niche space is reduced as species accumulate, eventually reaching some ecological limit (Rabosky 2009; Etienne et al. 2011; Etienne and Haegeman 2012). While plausible if contemporaneous taxa co-occur and compete for finite resources (Herrera-Alsina et al. 2018), this explanation for a decline in diversification with time seems less likely for non-adaptive radiations (Rundell and Price 2009; Czekanski-Moir and Rundell 2019). Some relatively young radiations in pond damselflies and other odonates have resulted from non-ecological speciation mechanisms, such as sexual selection or sexual conflict, and many closely related taxa are, therefore, only weakly ecologically differentiated from each other (McPeek and Brown 2000; Siepielski et al. 2010; Svensson 2012; Svensson et al. 2018). These features of damselfly communities suggest that niche partitioning does not strongly constrain speciation, although such weak niche differentiation might of course impact the longevity of newly formed lineages (see below). Moreover, our finding of abundant range reversals from warm-temperate to ancestral tropical biomes (Fig. 5) further argues against ecological diversity limits at a regional scale.

An apparent slowdown in diversification may also arise from statistical artifacts or sampling biases. For example, underparametrized models of molecular evolution can lead to underestimation of branch lengths, particularly in older branches of the tree (Revell et al. 2005). As diversification rate estimation ultimately relies on the distribution of branch lengths across the tree, this type of model misspecification can result in an artificial slowdown of diversification over time. An artificial slowdown can also be a product of incomplete lineage sampling, especially when taxon sampling is non-random (Cusimano and Renner 2010; Brock et al. 2011). However, in this study, we have used methods that explicitly incorporate sampling fractions of specific clades as a measure to counter this bias (Höhna 2015). Nonetheless, even when models are correctly specified, and sampling of extant taxa is accounted for, the “push of the past” can produce an apparent slowdown of diversification over time (Budd and Mann 2018). This survivorship bias occurs because we can only sample extant descendants from lineages that had high enough speciation rates as to have survived to the present. Deep branches in the tree that diversified more slowly and would have contributed to a more constant estimate of diversification rates over time are more likely to have gone extinct over the 105 Myr of Coenagrionoidea evolution, thereby leaving no descendants from which to infer their lower diversification rates. The “push of the past” remains a challenge to estimating temporal diversification dynamics when we lack information on whether and how many entire clades have gone extinct (Budd and Mann 2018).

The present distribution of pond damselflies, as many other clades, is characterized by higher species richness near the equator compared to temperate areas (Fig. 4) (Hillebrand 2004; Kinlock et al. 2018). This LDG in extant Coenagrionoidea is striking, considering that tropical lineages, particularly from India, South East Asia, and the Malaysian Archipelago, are probably underrepresented in the largest museum collections that contributed to this study. With this potential caveat in mind, our results indicated that none of the hypotheses based on accelerated speciation or reduced extinction in the tropics (see Introduction) is a likely explanation for why most extant damselfly species occur at low latitudes. While taking background diversification heterogeneity into account (Beaulieu and O’Meara 2016), we found similar rates of net diversification between the tropical and temperate regions of the globe (Fig. 3c). Nonetheless, speciation and extinction dynamics do tend to differ across latitudes, with both rates being highest in cold-temperate biomes, and thus canceling each other out. As a consequence, both warm-temperate and cold-temperate lineages tend to be relatively short-lived and replace one another at a faster rate than tropical lineages do, resulting in higher turnover rates outside of the tropics (Fig. 3d). Several studies have uncovered a similar pattern of increased lineage turnover in the temperate region or in harsher non-tropical environments (Weir and Schluter 2007; Botero et al. 2014; Pyron 2014; Harvey et al. 2020).

Faster lineage turnover at higher latitudes may come about in at least 2 ways. First, paleoclimatic fluctuations (e.g., DeConto et al. 2008) can cause the fragmentation of distribution ranges, a phenomenon that is particularly pronounced at higher latitudes (Jansson and Dynesius 2002). By repeatedly creating isolated refugia, climate fluctuations may have episodically fostered allopatric speciation (Weir and Schluter 2004; Milá et al. 2007; Sánchez-Ramírez et al. 2015; Morales-Barbero et al. 2018). However, the same recurrent climatic events can also increase extinction rates, either because range fragmentation reduces population size, or because hybridization and competitive exclusion drive extinction upon secondary contact (Dynesius and Jansson 2000; Barnosky 2005; Botero et al. 2014). Furthermore, climate fluctuations at higher latitudes might open up novel ecological opportunities that prompt speciation, by constantly renewing the availability of unoccupied niches (Schluter 2016). High environmental harshness in these novel environments at high latitudes might also result in the predominance of viability selection driven by abiotic environmental conditions, potentially increasing extinction risk and thereby accelerating lineage turnover (Haldane 1937; Gomulkiewicz and Holt 1995; Cutter and Gray 2016).

Whether it is driven by ecological factors (Cutter and Gray 2016; Schluter 2016), or non-ecological mechanisms (McPeek and Gavrilets 2006; Svensson et al. 2006; Siepielski et al. 2018), ephemeral speciation in temperate regions does not seem to have a net impact on diversification. Our results, therefore, highlight the role of the historical origin of Coenagrionoidea in the tropics as one of the main causes of the present-day LDG. This historical early start of diversification in the tropics has maintained the LDG despite an overall decrease in speciation rate (Fig. 2), and a net gain of lineages through biome shifts into temperate regions, particularly over the last 30 Myr (Fig. 4). Our findings that biome affinities strongly influenced ancestral distribution ranges, and that temperate lineages tend to return to the tropics (Fig. 5; Supplementary Fig. S42), suggest that dispersal out of the tropics has been limited by tropical niche conservatism, even in periods when warm and cold-temperate biomes were widely available. These results support previous studies finding stronger niche conservatism in tropical lineages (Smith et al. 2012; Pyron and Wiens 2013), and underscore how the potential for adaptive evolution into colder biomes is likely a primary factor governing the steepness of the gradient (Rangel et al. 2018).

As a result of an early start of diversification in the tropics, followed by similar net diversification rates across latitudinal regions, the Coenagrioniodea LDG is expected to persist, unless an external input of diversity to the temperate areas reverses this general pattern. Continuing the trend of the last 30 Myr, a trickle of tropical lineages reaching cold biomes could slowly contribute to a shallower LDG (Fig. 4). However, in a much shorter term, the smoothing of environmental gradients due to global warming (Loarie et al. 2009), might release adaptive constraints for lineages dispersing into higher latitudes. Examples of such rapid northward expansions have already been documented in Odonata (Paulson 2001; Grewe et al. 2013; Lancaster et al. 2015). The role of dispersal shaping the future steepness of the LDG thus warrants further investigation.

## Conclusion

The damselfly superfamily Coenagrionoidea originated in tropical areas, where most of its diversity is currently found (Fig. 1). An increasing number of studies have revealed the importance of both ecology and historical biogeography in producing regional differences in diversity across distinct clades of plants and animals (e.g., Rolland et al. 2014; Antonelli et al. 2015; Jablonski et al. 2017). Here, we have shown that in pond damselflies and their relatives, early tropical diversification followed by relatively slow shifts into warm-temperate and later cold-temperate biomes established the latitudinal diversity gradient observed today. Faster ecological speciation in temperate regions is unlikely to compensate for this time lag in the future, as speciation might be more ephemeral at higher latitudes (Fig. 3). However, climate change and lineage movements have also contributed to an increase of Coenagrionoidea diversity in cold biomes, currently at high latitudes (Fig. 4). Our results reveal a complex interplay between history, macroevolutionary processes, and dispersal shaping global diversity patterns.

## supplementary material

Data available from the Dryad Digital Repository: https://doi.org/10.5061/dryad.h18931znp
